# Could human chorionic gonadotropin modulate interleukin 1β to be a successful pregnancy predictor or not?

**DOI:** 10.5935/1518-0557.20200032

**Published:** 2021

**Authors:** Sedighe Amooee, Sara Davoodi, Leila Ghasmpour, Shaghayegh Moradi Alamdarloo, Ali Karimian, Jamshid Rahmati

**Affiliations:** 1 Infertility research center, Department of Obstetrics and Gynecology, School of Medicine, Shiraz University of Medical Sciences, Shiraz, Iran; 2 Maternal-fetal Medicine Research Center, Perinatology Department, Shiraz University of Medical Sciences, Shiraz, Iran; 3 Department of Physiology, School of Medicine, Shiraz University of Medical Sciences, Shiraz, Iran; 4 Department of Anesthesiology, School of Medicine, Shiraz University of Medical Sciences, Shiraz, Iran

**Keywords:** *in-vitro* fertilization, pregnancy outcome, human chorionic gonadotropin, interleukin 1β

## Abstract

**Objective:**

Reproductive medicine needs to find some ways to predict pregnancy outcomes and implantation, which are non-invasive and accurate. Immunologic factors and interleukins are good choices reported in the literature. The purpose of this study was to evaluate whether or not HCG administration can modulate interleukin 1β as a successful pregnancy predictor.

**Methods:**

This is a prospective cross-sectional study involving women with regular menstrual cycles who had frozen their embryos. They prepared their endometria with letrozole and human chorionic gonadotropin (HCG). Their interleukin 1β serum levels were checked on the day of HCG administration and embryo transfer. Its value assesses pregnancy outcome.

**Results:**

We had 44 women with mean age of 32.2±5.4, and clinical pregnancy rate of 31.8%, mean interleukin 1β before and after HCG injection in women who did not achieve pregnancy was 15.82±6.68pg/ml before HCG injection and 18.38±13.76pg/ml on the embryo-transfer day. It was high, but not significant (*p* value=0.210). In those participants who had clinical pregnancy before HCG injection, the mean interleukin 1β level was 17.29±7.00pg/ml and 29.72±10.41pg/ml on the day of embryo transfer, with significant changes (*p* value=0.001).

**Conclusion:**

HCG did increase the mean level of interleukin 1β, but it was not significant. High interleukin 1β level is a significant predictor of successful pregnancy in IVF cycles.

## INTRODUCTION

Early pregnancy prediction in infertile patients is a concern, which more than economic costs, it involves patients psychologically. Sometimes, limited frozen embryos is an acceptable reason for trying to transfer them in one cycle, which predicts a good outcome. The other indication for pregnancy prediction after embryo transfer is when couples prefer not to have multiple pregnancies, but they do not want to decrease success rates by transferring lower numbers of embryos ^(Ottosen et al., 2007)^.

Based on pregnancy and implantation physiology, there are some factors recommended in the literature that can help predict pregnancy. We designed a nomogram containing women’s age, progesterone and human chorionic gonadotropin (HCG) levels as predictors of success in pregnancy after embryo transfer ^(Kim et al., 2014)^. Immune cells like endometrial natural killer cells in the preimplantation endometrium represent a factor used to predict *in vitro* fertilization success ^(Kofod et al., 2017)^. It is important to choose a non-invasive method for this purpose so, some studies use ultrasound features of the endometrium, and uterine artery Doppler to predict pregnancy outcome ^(Ahmadi et al., 2017)^.

Immunologic factors and cytokine production patterns used for predicting pregnancy or complications have been reported in the literature ^(Perricone et al., 2012; Raghupathy & Szekeres-Bartho, 2016)^. Interleukin 1 is a factor associated with implantation rates and endometrium receptivity. Interleukin 1β is the cytokine most reported in studies, although there is no recommended cut-off value for it ^(Khadem et al., 2019; Kreines et al., 2018; Lekovich et al., 2015)^.

HCG triggers angiogenesis with endometrial stromal cells modulation with interleukin, and may lead to embryo implantation ^(Bourdiec et al., 2012)^. In other pregnancy situations, HCG is associated with increased interleukin 1β, such as to predict premature membrane rupture and chorioamnionitis ^(Tian et al., 2014)^.

In our study, we assessed the association between Interleukin 1β serum level before and after HCG triggering, and its association with pregnancy outcome. Our goal was to assess whether or not HCG administration can modulate interleukin 1β as a successful pregnancy predictor.

## MATERIALS AND METHODS

This study was a prospective cross-sectional study for a period of 6-months, starting in December 2018. It involved infertile women referred to the Infertility Center at Hazrate Zeinab Hospital, affiliated to the Shiraz University of Medical Sciences, and this study was approved through its Ethics Committee. The inclusion criteria were women 18-42 years old, having frozen embryos, normal uterine cavity, normal endometrium, BMI <35 kg/m^2^. The exclusion criteria were other maternal diseases, hydrosalpinx, and endometriosis. The women who signed the informed consent form and fulfilled the inclusion criteria participated in the study. The sample size was calculated to be 44 cases, with α=0.05 and power of 80%.

We evaluated the participants’ ovaries and endometrium with a transvaginal ultrasound on the second day of their cycles. They were prescribed 5 mg letrozole/day from the 3^rd^-7^th^ day of the menstrual cycle. We monitored their follicular development using vaginal ultrasonography, starting on the 10^th^ day of the menstrual cycle, if follicular diameter became ≥17mm and endometrial thickness reached 7-9mm, they were given 10000 units of HCG, and 36-48hour after that, 100mg progesterone intramuscular/day for 3 to 5 days based on embryo age. Then, the embryos were transferred, and the 100mg progesterone IM per day continued for 3 days after the transfer, and then they were changed to progesterone suppository 400 mg Q12hr (rectal or vaginal) 14-16 days after transfer.

If on the 10^th^ day the dominant follicle was ≥14mm to <17mm, then serial vaginal ultrasonography (every other day) was repeated without adding any medication until the follicle reached 17 mm or more, or the endometrial thickness reached 7-9 mm, then HCG was administered, and then it was continued. But if on the 10^th^ day the dominant follicle size was <14mm, human menopausal gonadotropin (HMG) was injected daily for 3-4 days, until the follicular diameter became >17mm or endometrial thickness became 7-9 mm; and then we injected the HCG. If by the 17^th^ day of the cycle, the dominant follicle did not reach 17 mm or endometrial thickness was less than 7 mm, the cycle was canceled.

We collected serum samples on the day of HCG injection and on the day of embryo transfer, after clot formation and they were centrifuged for interleukin 1 β, later stored at -80ºC and then we analyzed them using Eliza kits.

We measured their HCG level 14 days after embryo transfer and if it was more than 25IU/L, we defined it as a biochemical pregnancy. Ultrasonography was performed 28-30 days after embryo transfer, having fetal heartbeat was defined as a clinical pregnancy. Pregnant women were prescribed progesterone suppository 400mg twice daily, and it was continued until 12 weeks of gestational age.

We used SPSS statistics for data analysis. We compared the mean values using one-way analysis of variance (ANOVA) and two-sample *t*-tests. The proportions for the two groups were compared using the Wilcoxon Signed Ranks Test, *p*<0.05 was considered statistically significant.

## RESULTS

Table 1 depicts the demographic and hormonal data of the participants as mean ± SD (range). Among the participants, 31 women (70.5%) had primary infertility and 13 of them (29.5%) had secondary infertility. Table 2 shows the causes of infertility in the participants.

**Table 1:** Demographics and hormonal data of the participants

	Participants (n=44)
Age	32.20±5.40
BMI	25.48±3.50
FSH	5.66±1.93
AMH	3.53±2.85
TSH	2.54±1.18
Prolactin	16.21±10.28
AFC	14.55±6.08
Time of infertility	6.92±4.46

Data are presented as mean ± SD (range). FSH: Follicle-stimulating hormone; AMH: Anti-Müllerian hormone; BMI: body mass index; TSH: thyroid stimulation factor; AFC: antral follicular count.

**Table 2:** Causes of infertility in participants

	Participants (n=44)
Polycystic ovary syndrome	6 (13.6%)
Tubal factor	4 (9%)
Male factor	20 (45.4%)
Unexplained infertility	14 (31.8%)

Ovarian stimulation was carried out with GnRH agonist in four women, and 40 of them received GnRH antagonist. Other ovarian stimulation and embryo transfer cycles are reported on Table 3.

**Table 3:** *In-vitro* fertilization cycles data and pregnancy outcome

	Participants (n=44)
Gonadotropin dose	2531.25±1161.23
Follicles more than 15 mm	11.00±5,57
No. of oocytes in metaphase II maturation stage (MII)	8.89±5.15
Embryo number	5.59±3.65
Number of transferred embryos	2.59±0.72
Endometrial thickness on the day of transfer	8.23±0.40
Follicular phase days	12.64±1.96
3 days embryo	6 (13.6%)
5 days embryo	38 (86.4%)
Chemical pregnancy	15 (34.1%)
Clinical pregnancy	14 (31.8%)
Abortion	1 (2.3%)

According to the Wilcoxon Signed Ranks Test, the interleukin 1β mean values before and after HCG injection in the women who were not pregnant was 15.82±6.68pg/ml before HCG injection and 18.38±13.76pg/ml afterwards, respectively, on the day of embryo transfer. Despite its rise, it was not significant (*p* value=0.210). In participants who had clinical pregnancy, before and after HCG injection, the interleukin 1β mean level was 17.29±7.00pg/ml and 29.72±10.41pg/ml, respectively, on the day of embryo transfer, a significant change (*p* value=0.001).

## DISCUSSION

Our study illustrates that HCG injection can increase the mean level of interleukin 1 β, but it is not significant. There was a significant association between pregnancy and serum interleukin 1β rising. The exclusion criteria involved patients with endometriosis because of increasing interleukin 1β level in this disease ^(Lambert et al., 2014)^, and those with body mass index >35, because obesity seems to be the most important environmental factor affecting the onset and course of autoimmune diseases ^(Versini *et al*., 2014)^. We assessed ovarian stimulation protocols, GnRH agonist and antagonist in relation with interleukins ^(Aydin et al., 2014)^, but all the participants had frozen embryos and this factor did not affect the interleukin level.

It is true that some factors like age, ovarian reserve and semen parameters may predict ongoing pregnancy likelihoods in *in vitro* fertilization cycles (IVF) ^(Lintsen *et al*., 2007)^ but for reasons mentioned in the introduction, clinicians need predictors that are non-invasive and can predict pregnancy outcomes more accurately. ^Lekovich *et al*. (2015)^ used interleukin 1β and its antagonist receptor in late luteal phase for predicting ectopic pregnancy in IVF cycles. In another study, interleukin 1β helps predict premature rupture of membrane and chorioamnionitis ^(Tian *et al*., 2014)^. Despite these various situations that increase interleukin 1β illustrated here, this study should consider many confounding factors.

^Bourdiec et al. (2012; 2013)^ reported immunologic studies on early embryo implantation, that HCG modulates interleukin 1 as a factor has affects endometrial cell receptivity. Endogenous HCG effects on interleukins and interleukin 1β as predictors of pregnancy success stimulate studies like this, to find out whether or not exogenous HCG administration before embryo transfer cycles may increase interleukin. Increased interleukin 1β level induced by HCG is a reliable tip that in these women interleukin 1β is not a good factor for predicting pregnancy. In the present study, interleukin 1β rising due to exogenous HCG before and after, had not significant effect, although this theory is not confirmed.

## CONCLUSION

Based on the present study, HCG can increase the mean level of interleukin 1β, but it was not significant, its raise is a successful predictor of pregnancy. Other studies with larger samples are needed for concluding that HCG injection can increase pregnancy rates by increasing interleukin 1β.

## References

[r1] Ahmadi F, Akhbari F, Zamani M, Ramezanali F, Cheraghi R (2017). Value of Endometrial Echopattern at HCG Administration Day in Predicting IVF Outcome. Arch Iran Med.

[r2] Aydin Y, Hassa H, Isikci T, Colak O (2014). Follicular fluid and serum vascular endothelial growth factor, interleukin (IL)-1β and glycodelin concentrations: comparison between long-gonadotropin-releasing hormone (GnRH)-agonist and GnRH-antagonist cycles: a randomized controlled trial. Gynecol Endocrinol.

[r3] Bourdiec A, Shao R, Rao CV, Akoum A (2012). Human chorionic gonadotropin triggers angiogenesis via the modulation of endometrial stromal cell responsiveness to interleukin 1: a new possible mechanism underlying embryo implantation. Biol Reprod.

[r4] Bourdiec A, Calvo E, Rao CV, Akoum A (2013). Transcriptome analysis reveals new insights into the modulation of endometrial stromal cell receptive phenotype by embryo-derived signals interleukin-1 and human chorionic gonadotropin: possible involvement in early embryo implantation. PLoS One.

[r5] Khadem N, Mansoori M, Attaran M, Attaranzadeh A, Zohdi E (2019). Association of IL-1 and TNF-α Levels in Endometrial Secretion and Success of Embryo Transfer in IVF/ICSI Cycles. Int J Fertil Steril.

[r6] Kim JH, Jee BC, Suh CS, Kim SH (2014). Nomogram to predict ongoing pregnancy using age of women and serum biomarkers after in vitro fertilization cycles. Eur J Obstet Gynecol Reprod Biol.

[r7] Kofod L, Lindhard A, Bzorek M, Eriksen JO, Larsen LG, Hviid TVF (2017). Endometrial immune markers are potential predictors of normal fertility and pregnancy after in vitro fertilization. Am J Reprod Immunol.

[r8] Kreines FM, Nasioudis D, Minis E, Irani M, Witkin SS, Spandorfer S (2018). IL-1β predicts IVF outcome: a prospective study. J Assist Reprod Genet.

[r9] Lambert S, Santulli P, Chouzenoux S, Marcellin L, Borghese B, de Ziegler D, Batteux F, Chapron C (2014). Endometriosis: increasing concentrations of serum interleukin-1beta and interleukin-1sRII is associated with the deep form of this pathology. J Gynecol Obstet Biol Reprod (Paris).

[r10] Lekovich J, Witkin SS, Doulaveris G, Orfanelli T, Shulman B, Pereira N, Rosenwaks Z, Spandorfer SD (2015). Elevated serum interleukin-1β levels and interleukin-1β-to-interleukin-1 receptor antagonist ratio 1 week after embryo transfer are associated with ectopic pregnancy. Fertil Steril.

[r11] Lintsen AM, Eijkemans MJ, Hunault CC, Bouwmans CA, Hakkaart L, Habbema JD, Braat DD (2007). Predicting ongoing pregnancy chances after IVF and ICSI: a national prospective study. Hum Reprod..

[r12] Ottosen LD, Kesmodel U, Hindkjaer J, Ingerslev HJ (2007). Pregnancy prediction models and eSET criteria for IVF patients--do we need more information?. J Assist Reprod Genet.

[r13] Perricone C, de Carolis C, Perricone R (2012). Pregnancy and autoimmunity: a common problem. Best Pract Res Clin Rheumatol.

[r14] Raghupathy R, Szekeres-Bartho J (2016). Dydrogesterone and the immunology of pregnancy. Horm Mol Biol Clin Investig.

[r15] Tian CF, Lv FH, Wang M, Gu XS (2014). Serum β-human chorionic gonadotropin and interleukin-1 as diagnostic biomarkers for the premature rupture of membranes and chorioamnionitis. Biomed Rep.

[r16] Versini M, Jeandel PY, Rosenthal E, Shoenfeld Y (2014). Obesity in autoimmune diseases: not a passive bystander. Autoimmun Rev.

